# Adaptive vs. non-adaptive cognitive training by means of a personalized App: a randomized trial in people with multiple sclerosis

**DOI:** 10.1186/s12984-016-0193-y

**Published:** 2016-10-04

**Authors:** Ludovico Pedullà, Giampaolo Brichetto, Andrea Tacchino, Claudio Vassallo, Paola Zaratin, Mario Alberto Battaglia, Laura Bonzano, Marco Bove

**Affiliations:** 1 Department of Experimental Medicine, Section of Human Physiology, University of Genoa, Genoa, Italy; 2 Italian Multiple Sclerosis Foundation, Scientific Research Area, Genoa, Italy; 3 Department of Life Science, University of Siena, Siena, Italy; 4 Department of Neuroscience, Rehabilitation, Ophthalmology, Genetics, Maternal and Child Health, University of Genoa, Genoa, Italy

**Keywords:** Application software, Cognitive rehabilitation, Adaptive working load, Personalized treatment, Multiple sclerosis

## Abstract

**Background:**

Cognitive impairment is common in multiple sclerosis (MS), but the definition of the best cognitive rehabilitation tools and features is still an open issue among researchers. The aims of the present study were to evaluate the effectiveness of COGNI-TRAcK (a customized application software delivering personalized working memory-based exercises) on cognitively impaired people with MS and to investigate the effects of an adaptive vs. a non-adaptive cognitive training administered by means of COGNI-TRAcK.

**Methods:**

Twenty eight patients (20 women, age 47.5 ± 9.3 years, Expanded Disability Status Scale score 3.8 ± 1.9) were randomized in two homogeneous groups, both performing a 8-week home-based cognitive rehabilitation treatment by means of COGNI-TRAcK. The study group (ADAPT-gr) underwent an adaptive training given by the automatic adjustment of tasks difficulty to the subjects’ performance, whilst the control group (CONST-gr) was trained at constant difficulty levels. Before and after the treatment, patients’ cognitive status was assessed using a gold standard neuropsychological evaluation. Moreover, the mostly affected cognitive domains in MS (i.e., attention, concentration and information processing speed) were also assessed 6 months after the end of the treatment.

**Results:**

The analysis of variance showed a significant Group*Time interaction in six out of ten tests of the cognitive evaluation. Post-hoc analysis revealed a significant improvement between the performances before and after the intervention only in the ADAPT-gr in tests evaluating verbal memory acquisition (*p* <0.05) and delayed recall (*p* = 0.001), verbal fluency (*p* = 0.01), sustained attention, concentration and information processing speed (*p* < 0.01). This last effect was maintained also after 6 months (*p* < 0.05).

**Conclusions:**

We concluded that COGNI-TRAcK represents a suitable tool to administer a personalized training to cognitively impaired subjects and that an adaptive working load is a crucial feature determining the effectiveness of cognitive treatment, allowing transfer effects to several cognitive domains and long-term maintenance of results.

## Background

Multiple sclerosis (MS) is a chronic immune mediated disease of the central nervous system characterized by the presence of widespread lesions in the brain and spinal cord, resulting in a broad range of symptoms [1]. Cognitive impairment (CI) is frequent among people with multiple sclerosis (PwMS), with prevalence rates ranging from 43 to 70 % [2]. The most commonly affected domains are new learning and memory [3], attention [4], information processing speed [5] and executive functions [6]. Impairments in these functions often lead to reduced occupational profile, social participation, and quality of life among PwMS [7]. Alleviation of deficits on cognitive functioning is the main goal of cognitive rehabilitation (CR), and research should be addressed to characterize the best way to administer CR to patients. In the last two decades, different studies have been produced aiming at validating computer-based CR programs in order to supply effective, usable and accessible tools preventing from the constraints of face-to-face interventions (such as the cost of personnel or the patients/operators mobility). Results in this fields showed that computer-based cognitive training can improve cognitive functions in elderly [8] and in people with stroke [9] or Alzheimer’s disease [10].

With regards to MS, although recent works showed that computer-assisted CR can improve some aspects of cognitive deficits [11–14], research on this field produced overall equivocal results [15–18], arising the need for further investigations [19]. In particular, the issue about what treatment features are the most effective remains unsolved. For example, Solari et al. supposed that a domain-specific training would be more effective than a non-specific one, but they reported no differences between the two interventions [17]. Also, the target of the intervention (domain-specific approach or more general cognitive stimulation) is an open issue among researchers. Although the traditional approach to CI in PwMS involved learning and memory-based interventions, recently the focus has moved to other domains such as executive functions and attention [19]. It is known that working memory (WM), the limited capacity storage system involved in the maintenance and manipulation of information over short periods of time [20], is strictly linked to attention [21] and underlies a wide range of higher-order cognitive activities. Moreover, recent works showed that an intensive training based on WM exercises seems to positively influence several cognitive functions in different populations [22–25]. In a review on this issue, Takeuchi and colleagues described the most used methodologies for WM training, focusing on the factors that may influence the training efficacy [26]. Among the cited characteristics, adaptability (i.e., the adjustment of the task difficulty depending on the subject’s performance) and intensiveness of training (i.e. how the same amount of training is massed into shorter or longer time of periods) are mentioned as fundamental features enhancing the effects of the administered intervention. Thus, we hypothesized that an adaptive working load, coupled with intensiveness of interventions, could be crucial in determining the actual effectiveness of the treatment.

For these reasons, we recently developed and validated on PwMS an application software (App) for portable devices, named Cognitive Training Kit (COGNI-TRAcK), administering a user-friendly and personalized treatment based on WM exercises [27]. The App can automatically adapt the exercises difficulty levels to the user’s maximal working threshold depending on his/her performance. Moreover, it can be easily used at home enhancing the possibility to schedule an intensive training and ensuring adherence to treatment.

Therefore, the aim of the present study was to evaluate the effectiveness of an intensive and adaptive training based on WM exercises in improving the cognitive status of PwMS. Towards this goal, we delivered CR through COGNI-TRAcK to a study group trained with adaptive WM tasks and to a control group trained with non-adaptive WM tasks. We compared the effect of the intervention on the basis of the results obtained by the two groups on a gold standard neuropsychological evaluation. As strongly suggested in a recent critical review [28], an “active” control group was designed in our study, in order to identify potential positive effects of an adaptive working load compared to a non-adaptive treatment.

## Methods

### Patients

We considered for eligibility outpatients referred to the Italian MS Society (AISM) Rehabilitation Centre of Genoa (Italy) who complained of poor memory or attention. Out of 37 screened patients, 32 accepted to participate and 30 met the MS diagnostic criteria of McDonald et al. [29] and were in a stable phase of the disease (i.e., no relapses in the last 3 months). These patients’ cognitive status was evaluated by means of the Rao’s Brief Repeatable Battery of Neuropsychological Tests (BRB-NT) [30], and we considered as inclusion criterion a score at least 1.5 standard deviation (SD) below the mean normative values at one or more components of the BRB-NT. Exclusion criteria were: age less than 18, one or more exacerbations in the 3 months prior to enrolment, ongoing major psychiatric disorder, benzodiazepines or antidepressants assumption, severe visual loss, dyscalculia or acalculia.

**Fig. 1:**
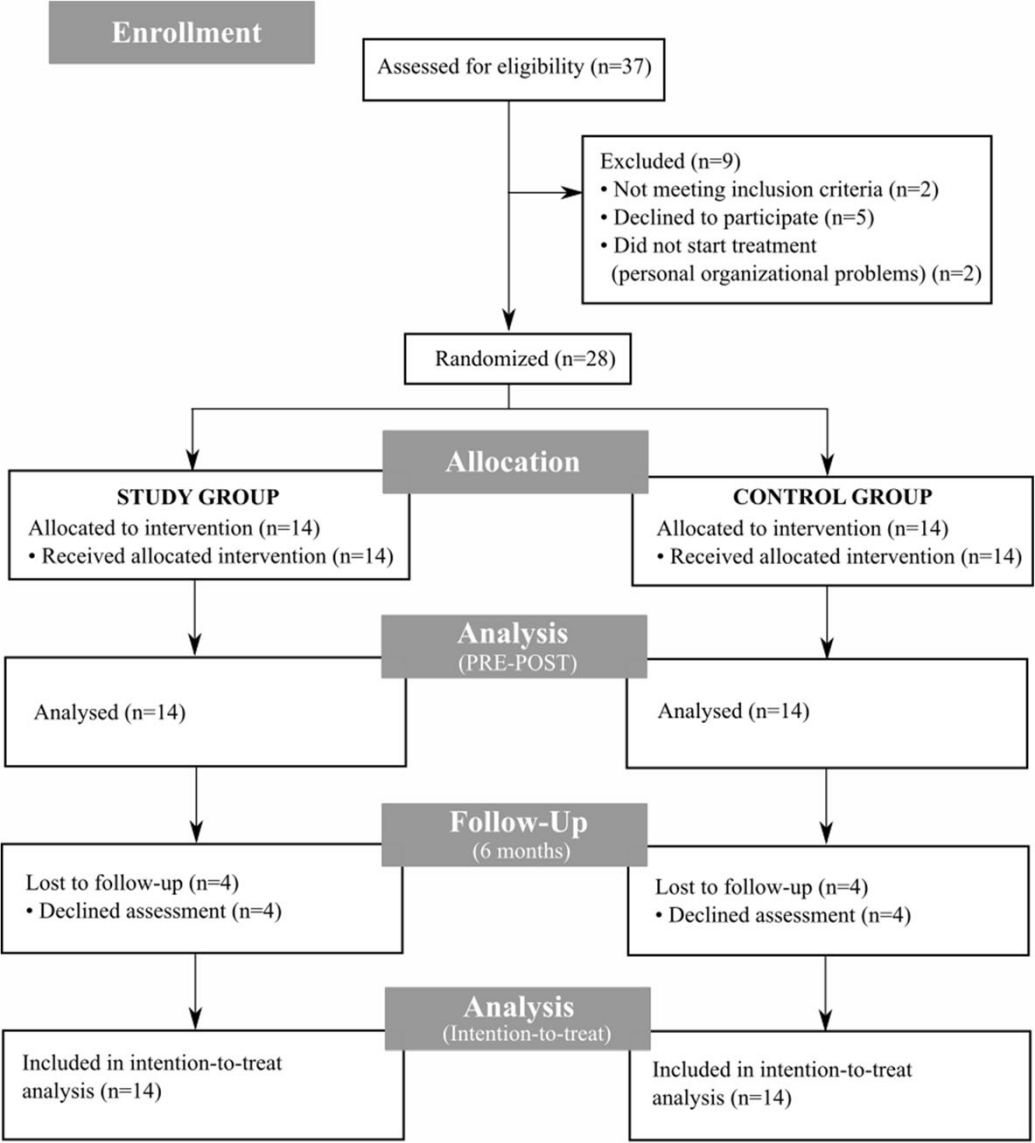
Participants flow diagram. Flowchart illustrating patients’ participation. Number of patients screened, included and considered for analysis is specified. Also, number of and reasons for exclusion and dropout is reported

Two enrolled patients did not start the rehabilitation treatment because of personal issues (work and family demands onset). Thus, twenty-eight PwMS (20 women and 8 men) received the allocated intervention and were considered in the analysis (see Fig. 1 for the flow diagram showing excluded patients and dropouts).

Since eight patients concluded the rehabilitative intervention and the assessments at baseline and after the treatment, but were lost at follow-up (i.e., after 6 months), an intention-to-treat analysis was used when examining follow-up data in order to preserve the benefit of randomisation [31] (see Statistical Analysis section for details).

Mean age of PwMS included in this study was 47.5 ± 9.3 years (range 28–67), and mean education was 11.7 ± 3.4 years. Mean age at disease onset was 30.2 ± 11.2 years, mean disease duration was 14.5 ± 9.4 years and mean Expanded Disability Status Scale (EDSS) score was 3.8 ± 1.9. Among the subjects, 17 were affected by a relapsing–remitting (RR) and 11 by a secondary progressive (SP) MS course. Eighteen of them were treated with disease-modifying drugs (DMD). The enrolled patients were randomly assigned by a blinded psychologist to the study group or to the control group (see next section). The groups did not differ for any demographic or clinical characteristic (Table 1).

**Table 1:** Characteristics of the study sample

	Total	Study group	Control group	*p*
Gender female/male	20/8	9/5	11/3	0.40^a^
Mean age (years, SD)	47.5 (9.3)	49.0 (7.1)	46.1 (11.2)	0.47^b^
Mean education (years, SD)	11.7 (3.4)	12.8 (3.1)	10.7 (3.5)	0.14^b^
Mean age at onset (years, SD)	30.2 (11.2)	29.1 (8.2)	31.29 (13.8)	0.63^b^
Mean disease duration (years, SD)	14.5 (9.4)	16.6 (8.6)	10.4 (6.6)	0.31^b^
Disease course RR/SP	17/11	8/6	9/5	0.70^a^
EDSS (mean, SD)	3.8 (1.9)	3.6 (1.6)	4.1 (2.3)	0.55^b^
Treatment with DMD (#, %)	18 (64.2)	8 (57.1)	10 (71.4)	0.43^a^

*Abbreviations*: *SD* standard deviations, *RR* relapsing-remitting, *SP* secondary progressive *DMD* disease-modifying drugs

Reported *p* values refer to: ^a^Pearson’s Chi Square test for categorical variables; ^b ​﻿^Student's t-test for continuous variables

All recruited patients provided a written informed consent according to the Declaration of Helsinki [32], and the study was approved by the Ethics Committee of Azienda Ospedaliera San Martino, Genoa, Italy.

### Intervention

All participants executed a 8-week training consisting of five 30-min sessions a week self-administered at home by means of COGNI-TRAcK. We used COGNI-TRAcK since it is well accepted by patients with MS, and particularly suitable in order to deliver intensive, automatically adaptive and monitored cognitive training [27].

**Fig. 2:**
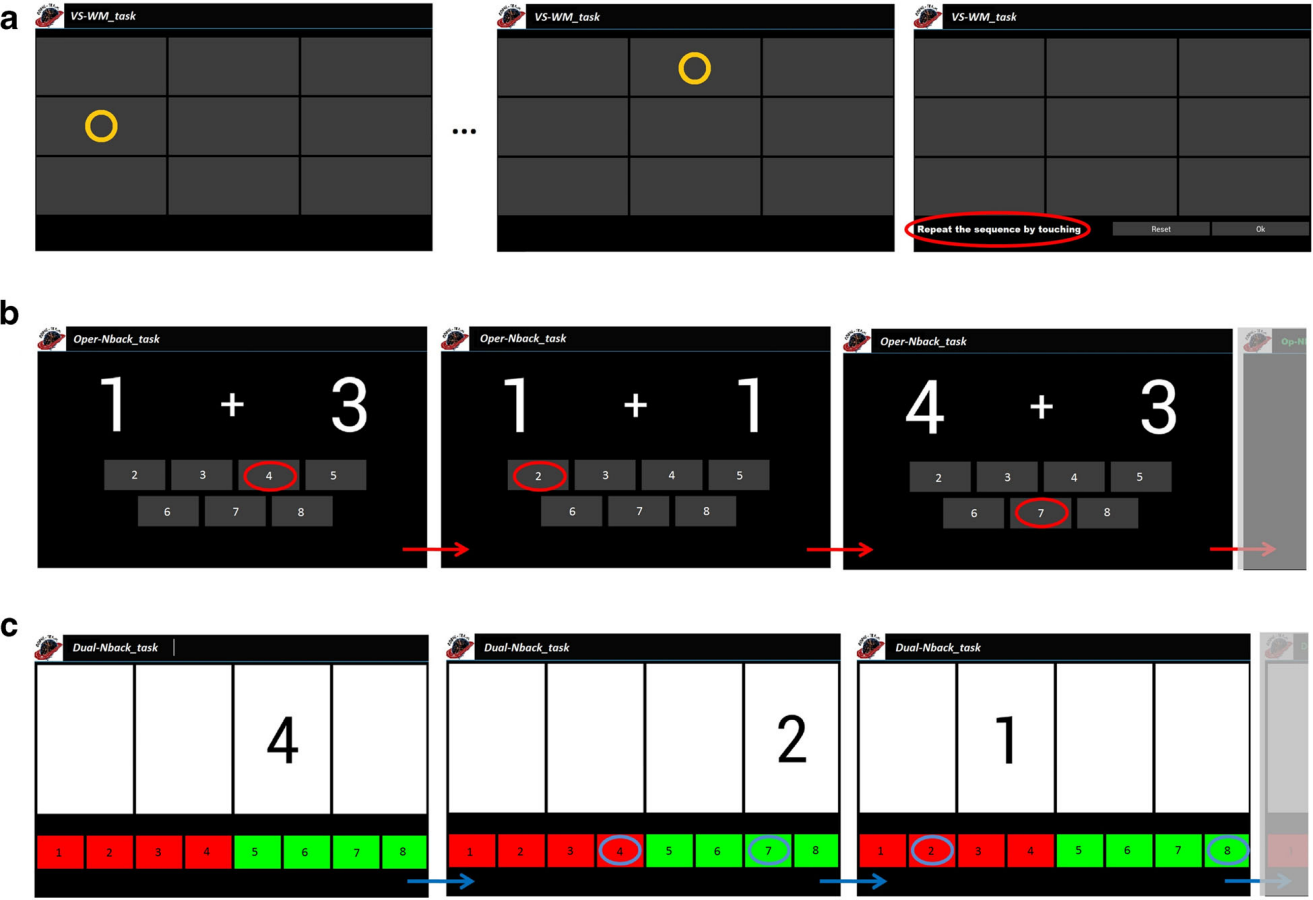
COGNI-TRAcK working memory-based exercises. The three exercise types implemented by COGNI-TRACK. In the VS-WM_task (**a**) patients had to remember a random sequence of visual stimuli presented one at a time in a grid-like interface and correctly reproduce it by touching the corresponding locations on the screen. In Oper-Nback_task (**b**), a sequence of pair of numbers was presented on the screen and patients were asked to memorize the sum and to push the button on the keyboard that corresponded to *N* previous stimuli (*N* back rule). If *N* = 0, patients had to touch the button corresponding to the current sum (as illustrated by circles in **b**). When the difficulty level increased, i.e., *N* = 1 (or higher), patients had to answer the correct result deferred by one (or more) new pairs. In the Dual-Nback_task (**c**) the stimuli consisted of numbers, 1 to 4, presented in one of four cells on a line. Patients were asked to memorize the identity and location of the stimuli. Then, they had to push the buttons on the left of the keyboard to indicate the identity and the buttons on the right of the keyboard to indicate the location of the stimuli. As for the Oper-Nback_task, patients were asked to answer according to the *N*-back rule, i.e., pushing the correct answer deferred by *N* new stimuli (in **c** an example of the correct answers for *N* = 1 is given)

COGNI-TRAcK implements three different types of exercises (here, each one executed for about 10 min a session) which were shown to be effective in improving the cognitive status in healthy subjects [25]. The exercises consisted in: (i) a visuospatial WM task (VS-WM_task); (ii) an “operation” *N*-back task (Oper-Nback_task); (iii) a “dual” *N*-back task (Dual-Nback_task). A detailed description of the exercise types is reported in a previous work [27] and is presented in Fig. 2.

The possibility to set the option to automatically adapt the exercises difficulty level on the users performance was the key feature chosen in our study in order to discriminate the type of intervention delivered to the two groups of patients. Indeed, the study group (ADAPT-gr) received an adaptive training, whilst the control group (CONST-gr) received a non-adaptive training (i.e., constant difficulty level). In detail, the adaptive training was structured so that the exercises difficulty level increased by one step every time the user performed a correct exercise. On the other hand, the difficulty level decreased by one step if the exercise was incorrect for three times in a row. Conversely, the CONST-gr followed a non-adaptive training, consisting in an algorithm implementing two low difficulty levels alternating every day regardless of the user’s performance (Table 2). This precaution was adopted in order to prevent a possible un-motivating effect due to the continuous execution of the same task along the rehabilitation period and to avoid loss of adherence to the training as much as possible.

**Table 2:** Modifiable parameters of the exercises. Minimum and maximum values of each exercise type parameters are reported for both groups

Exercise type	Parameter	ADAPT-gr	CONST-gr
		Min; Max	Min; Max
VS-WM_task	Number of stimuli Frequency	4; 7 1 stim/2 s; 1 stim/s	4; 5 1 stim/2 s (constant)
Oper-Nback_task	*N* parameter Number of stimuli Frequency	0; ∞ 5; (*N* + 1)*5 1 stim/5 s; 1 stim/3 s	0; 1 5; 10 1 stim/5 s (constant)
Dual-Nback_task	*N* parameter Number of stimuli Frequency	0; ∞ 5; (*N* + 1)*5 1 stim/5 s; 1 stim/3 s	0; 1 5; 10 1 stim/5 s (constant)

*Abbreviations*: *VS-WM_task* visuospatial working memory task, *Oper-Nback_task* “operation” N-back task, *Dual-Nback_task* “dual” N-back task, *ADAPT-gr* adaptive training group *CONST-gr* constant training group

For each type of exercise, both groups started from the same difficulty level. Only for the ADAPT-gr, the training algorithm was set so that the difficulty level was increased modifying the frequency at which stimuli were presented and task-specific parameters, namely the number of visual stimuli of a sequence to remember for the VS-WM_task, and the *N* parameter (i.e., number of previously observed stimuli to refer giving the answers, according to standard *N-*back tasks rule) for the Oper-Nback_task and the Dual-Nback_task. *N* parameter determines the number of stimuli for these two exercise types, following the mathematical rule (*N* + 1)*5 (Table 2).

### Outcome assessment

Patients’ adherence to treatment was calculated as the percentage of completed training sessions out of the total number of scheduled sessions (100 % corresponded to the 40 sessions), according to a previous work [27]. The percentage of correct executed exercises and the mean difficulty level maintained during the training was automatically recorded by COGNI-TRAcK for each patient. A detailed report was then generated and exported for further analysis.

A complete neuropsychological assessment was performed at baseline, i.e., before the rehabilitative treatment (PRE) and at the end of the treatment (POST) for both groups. A subset of the complete assessment was also performed 6 months after the end of the treatment in order to test the long-term effect of the intervention at follow-up (FU). We did not evaluate PwMS cognitive performance with the complete neuropsychological assessment at FU in order to minimize the patients’ obligation and maximise adherence.

The complete neuropsychological assessment included the following measures: the BRB-NT and the Wisconsin Card Sorting Test (WCST) [33]. The BRB-NT assesses the most frequently impaired cognitive domains in PwMS and incorporates the following tests: Selective Reminding Test, for verbal memory acquisition (Selective Reminding Test-Long Term Storage, SRT-LTS; Selective Reminding Test-Consistent Long Term Retrieval, SRT-CLTR) and delayed recall (Selective Reminding Test-D, SRT-D); 10/36 Spatial Recall Test (SPART), for visual memory acquisition and delayed recall (Spatial Recall Test-Delayed, SPART-D); Paced Auditory Serial Addition Test in its variations at different stimuli presentation speed (PASAT-3 and PASAT-2) and Symbol Digit Modalities Test (SDMT), for sustained attention, concentration and information processing speed; Word List Generation (WLG), for verbal fluency on semantic stimulus. The WCST was included in the assessment since it is one of the most used tasks to investigate frontal lobe executive functions, which are underrepresented in the BRB-NT.

The subset of scales used for FU evaluation included PASAT-3 and SDMT since they are tests investigating information processing speed, which was shown to be not only the cognitive domain most widely affected by MS but also the first cognitive deficit to emerge in MS [34]. Moreover, PASAT is part of the Multiple Sclerosis Functional Composite, developed to measure impairment and disability in MS [35], and it is widely used in clinical research. Besides, it was recently suggested that SDMT can represent by itself a useful screening tool to measure CI in MS since it showed high sensitivity and specificity in predicting the outcome of a complete neuropsychological test battery [36, 37].

Normative values and correction factors used in the present study refer to the Italian validation of the BRB-NT, published by Amato et al. [38].

### Statistical analysis

We analyzed differences between the groups regarding demographic and clinical variables using Student’s t-test for independent samples for continuous data, while categorical variables were compared by Pearson’s Chi Square test.

Cognitive performance at baseline was compared between the two groups by means of a Student’s t-test for independent samples applied to each test of the BRB-NT.

Concerning the results of the CR intervention, an analysis of variance (ANOVA) was carried out. In particular, correct data of each test were entered in a two-way ANOVA considering the Group (ADAPT-gr or CONST-gr) as between-subject factor and Time of assessment (PRE and POST) as within-subject factor. Moreover, for the tests performed at follow-up, we considered Time of assessment as within-subject factor at three levels (PRE, POST and FU). For this analysis, missing data due to dropouts after 6 months were replaced by the last known value before the participant was lost (here, the score obtained at the POST session), according to the “last observation carried forward” (LOCF) analysis. LOCF analysis is one of the most commonly used methods for the imputation of missing values in the analysis of continuous outcomes [39].

All post hoc pairwise comparisons were performed using the Newman-Keuls test. For all analysis conducted, *p* values below 0.05 were considered significant.

## Results

### Training

Both groups trained along the 8 weeks without reporting any complaint. Overall adherence to treatment was 87 % and no difference was found between the two groups (*t* = 0.24; *p* = 0.81).

As a consequence of the set algorithm, ADAPT-gr and CONST-gr trained at different mean levels. This produced in turn different performance obtained by the two groups in the three WM exercises. In particular, concerning the VS-WM_task, the mean difficulty level at which ADAPT-gr trained was characterized by a number of stimuli of 6.24 ± 0.61 and by an inter-stimulus interval of 1.44 ± 0.13 s. The mean percentage of correct VS-WM_task exercises performed by ADAPT-gr was 24.50 ± 6.46 %. The non-adaptive algorithm followed by CONST-gr maintained a mean number of stimuli of 4.46 ± 0.02 and a constant inter-stimulus interval of 2.0 s. The mean percentage of correct VS-WM_task exercises performed by CONST-gr was 71.55 ± 13.85 %. With regards to the Oper-Nback_task, ADAPT-gr trained at a mean difficulty level characterized by a *N* parameter value of 2.16 ± 0.64 and a inter-stimulus interval of 4.13 ± 0.20 s, and obtained a mean correct tasks percentage of 26.43 ± 5.11 %. The CONST-gr trained at mean *N* parameter value of 0.36 ± 0.02 and at constant stimuli presentation rate (1 stimulus every 5 s), and obtained a mean correct tasks percentage of 61.56 ± 25.78 %. Finally, the ADAPT-gr trained at mean Dual-Nback_task difficulty level characterized by *N* parameter value of 1.55 ± 0.48 and inter-stimulus interval of 5.20 ± 0.57 s, and obtained a mean correct tasks percentage of 30.20 ± 6.42 %. The CONST-gr trained at mean 0.37 ± 0.02 *N* parameter value and at constant 5.0 s inter-stimulus interval. They obtained a mean correct task percentage of 63.08 ± 27.57 %.

### Neuropsychological assessment

The two groups did not differ in the cognitive measures at baseline, except for one test of the BRB-NT. In detail, only the SRT-LTS score was significantly higher in the CONST-gr with respect to the ADAPT-gr (*t* = 2.10, *p* = 0.045).

Regarding the results obtained by the two groups at the neuropsychological battery after the intervention, the two-way ANOVA showed an effect of Time (PRE vs. POST) for all the tests. Moreover, in 6 out of 10 tests, an effect of the interaction Group*Time was found. In detail, only an effect of Time was observed in SRT-LTS (*F* = 19.37, *p* = 0.0001), SPART (*F* = 13.38, *p* = 0.001), SPART-D (*F* = 11.09, *p* = 0.002), and WCST (*F* = 13.77, *p* = 0.001), whilst an effect of the interaction Group*Time was observed in SRT-CLTR (*F* = 4.40, *p* < 0.05), SDMT (*F* = 8.92, *p* < 0.01), PASAT-3 (F 0 15.04, *p* < 0.001), PASAT-2 (*F* = 14.99, *p* < 0.001), SRT-D (*F* = 12.01, *p* = 0.001) and WLG (*F* = 6.67, *p* = 0.01). For all these tests, post hoc analysis revealed that the ADAPT-gr obtained a score significantly higher after the intervention with respect to baseline (see Table 3 for statistical details), while the CONST-gr obtained PRE and POST similar scores.

**Table 3:** Performance on the neuropsychological battery of the two groups (ADAPT-gr and CONST-gr) before (PRE) and after (POST) the cognitive rehabilitative intervention

Test	CONST-gr		ADAPT-gr		*p* values
time	PRE	POST	PRE	POST	
SRT-LTS	32.14 (10.43) (18.77–54.17)	38.33 (15.13) (10.96–60.17)	24.59 (8.45) (6.55–41.56)	39.79 (11.75) (5.55–52.16)	NS
SRT-CLTR	19.98 (10.92) (5.44–42.12)	23.50 (15.97) (2.16–51.78)	15.80 (10.01) (1.91–32.62)	31.08 (14.00)* (3.91–53.24)	**0.003**
SPART	13.64 (4.91) (3.94–23.56)	15.99 (3.70) (10.94–24.62)	13.82 (4.58) (8.31–23.78)	19.13 (5.46) (10.31–28.15)	NS
SDMT	36.70 (9.54) (21.24–50.53)	38.08 (9.09) (19.24–51.53)	39.10 (11.60) (22.24–61.38)	46.03 (11.52)* (24.27–64.38)	0.0001
PASAT-3	33.43 (8.74) (20.08–46.06)	32.99 (9.80) (15.38–51.53)	28.11 (14.15) (–1.02–43.68)	44.63 (13.60)* (8.19–60.38)	**0.0002**
PASAT-2	22.17 (8.98) (3.79–31.10)	24.12 (8.85) (11.79–43.10)	18.10 (9.83) (–0.67–35.45)	33.07 (11.12)* (6.87–47.45)	**0.0002**
SRT-D	6.08 (2.54) (2.48–10.88)	6.71 (3.19) (1.48–12.88)	5.37 (1.88) (1.87–7.88)	8.96 (2.18)* (5.87–14.08)	**0.0002**
SPART-D	4.16 (2.48) (–0.72–9.59)	5.30 (1.31) (3.28–7.67)	4.56 (1.22) (3.18–7.92)	6.15 (2.10) (2.92–10.18)	NS
WLG	33.21 (10.88) (7.88–64.88)	35.36 (12.73) (15.88–50.88)	38.15 (5.96) (23.88–49-12)	45.38 (7.37)* (27.88–55-12)	**0.0002**
WCST	2.50 (1.74) (0–5)	3.00 (1.57) (0–5)	3.15 (1.46) (0–5)	4.23 (1.36) (2–6)	NS

*Abbreviations*: *SRT-LTS* selective reminding test-long term storage, *SRT-CLTR* selective reminding test-consistent long-term retrieval, *SPART* 10/36 spatial recall test, *SDMT* symbol digit modalities test, *PASAT-3/-2* paced auditory serial addition test, *SRT-D* selective reminding test-delayed, *SPART-D* 10/36 spatial recall test-delayed, *WLG* word list generation on semantic stimulus, *WCST* Wisconsin card sorting test, *CONST-gr* constant group, *ADAPT-gr* adaptive group

Mean (SD, *range*) scores are reported for each test. Listed *p* values refer to post hoc comparison between PRE and POST results of ADAPT-gr where an effect of Group*Time was found

### Follow-up

**Fig. 3:**
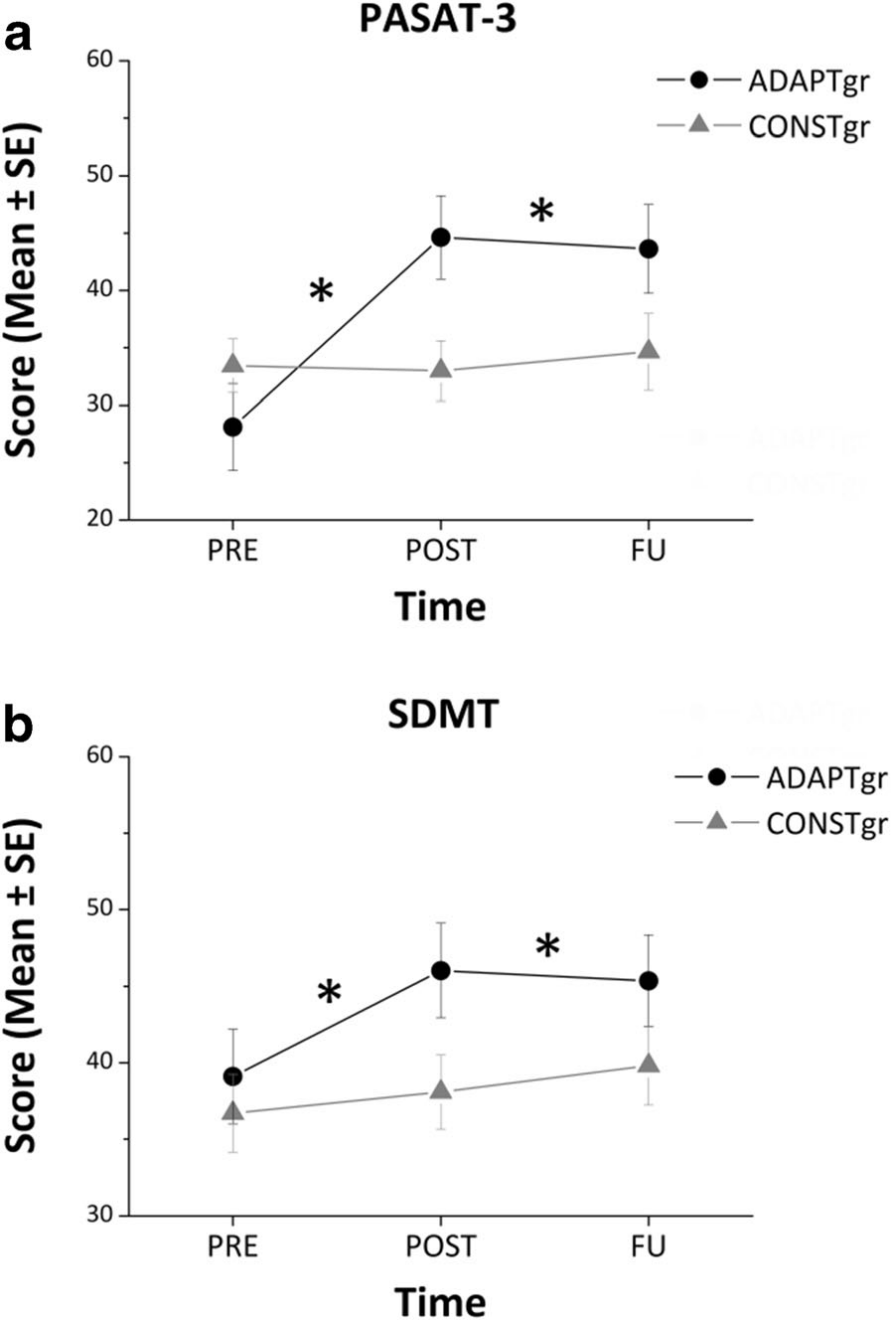
Follow-up results. Results obtained by the two groups before (PRE), immediately after (POST) and 6 months after (FU) the cognitive rehabilitation intervention at PASAT-3 (**a**) and SDMT (**b**). Missing data at FU were replaced by means of LOCF analysis. * refers to post hoc analysis *p* values < 0.005. Abbreviations - SE: Standard Error

Twenty patients (10 ADAPT-gr and 10 CONST-gr subjects) concluded the follow-up assessment performed by means of PASAT-3 and SDMT; LOCF analysis was used to replace missing data. The two-way ANOVA showed a significant interaction of Group*Time for both tests (*F* = 9.69, *p* < 0.001 for PASAT-3; *F* = 3.50, *p* < 0.05 for SDMT). In particular, post hoc analysis revealed that the ADAPT-gr obtained higher scores after the cognitive rehabilitative intervention and 6 months after the end of the training compared to baseline at both PASAT-3 and SDMT, whilst the CONST-gr performance did not change across time (Fig. 3).

## Discussion

In the present study, we investigated the effects of a CR intervention based on WM exercises comparing the impact of an adaptive and a non-adaptive working load on cognitively impaired PwMS. In particular, we aimed at defining some crucial features of the intervention affecting the outcome in terms of cognitive functions. For this reason, we used the customized App COGNI-TRAcK [27], designed to deliver intensive, monitored and personalized training based on WM exercises. The App allows low-cost training at home with off-the-shelf devices, ensuring several advantages with respect to other computer-based tools previously reported in the literature. In fact, many studies relied on an ambulatory setup [11, 14, 15, 17], and this design may lead to high refusal rate (about 22 %, when reported [17]) due to the need to travel to the rehabilitation centre. On the other hand, home-based studies referred low dropout rate (about 11 % [12, 13]), but failed to report objective data on adherence to the cognitive training. In our case, the CR program by means of COGNI-TRacK yielded low refusal and dropout rate and high level of adherence to the treatment, despite of the intervention intensiveness. Moreover, the most of the studies on CR utilized training programs produced by a third party which are not always freely available [11, 12, 14, 17], or required that the participants owned a home computer and had access to broadband internet services [40].

Taken together, all these considerations suggest that the tool and design used in the present study can be easily translated into clinical practice and assistance of a large number of patients.

Another crucial aspect of this study is that COGNI-TRAcK allows to set adaptive and non-adaptive algorithms and we used this options to differently train the study group (ADAPT-gr) and the control group (CONST-gr). In this way, the two groups interventions differed only for the implemented algorithm whereas the tool used for the training, the exercise types, and the intensiveness of the intervention were the same for all the patients involved. This study design was adopted following recent directions about the evidence for training-dependent neuroplasticity [28], suggesting that more reliable results should be obtained comparing groups who have been trained on different task features instead of comparing treated with waiting list subjects. In fact, many CR studies conducted in PwMS compared results obtained by the study group with a control group who received either no intervention [11, 12] or non-specific placebo treatments [14], which may lead to find changes due to a general effect of any training.

Concerning the efficacy of the cognitive training, results showed a significant improvement between PRE and POST only for the ADAPT-gr in six out of ten tests. These results provide evidence that an adaptive exercise load, ensuring that the patient is training at his/her maximal working threshold, is more effective than a non-adaptive training in improving cognitive functions. Moreover, the wide spread of scores obtained by participants at baseline (as shown by the range of BRB-NT scores reported in Table 3) suggests that the App is useful for many types and severities of impairment.

These findings are in line with recent works on PwMS that showed some beneficial effects of rehabilitative interventions on the patients’ cognitive status [11, 14, 40]. Among the studies that involved an “active” control group, Hancock et al. showed that an adaptive treatment based on processing speed and WM was able to improve the targeted functions in PwMS [40]. However, the authors did not find a transfer effect to all other, associated cognitive skills. Instead, an adaptive training based on WM exercise by means of COGNI-TRAcK seems to elicit a transfer effect on non-trained cognitive domains. In fact, an improvement of the ADAPT-gr was observed not only in the tests specifically investigating the training-related domains (i.e., attention and information processing speed), such as PASAT and SDMT, but also in the tests evaluating new learning and verbal memory (SRT-CLTR and SRT-D) and verbal fluency (WLG). The difference between our and previously reported results may be due to the different tests used to evaluate the cognitive domains or to the general approach of the training, suggesting that intensive and adaptive WM training is an excellent way to improve several cognitive domains.

Further, we did not find a significant interaction Group*Time, but an effect of Time in the tests investigating visuospatial memory and delayed recall (SPART and SPART-D). This could be due to the slight difference between the two groups in the difficulty levels adaptation of the VS-WM_task. In fact, the CONST-gr training ranged between 4 and 5 stimuli, and the ADAPT-gr trained at a maximum of 7 stimuli. This limitation was set in order to avoid stress in the patients facing an excessively challenging task. In fact, almost all patients referred that the VS-WM_task was the most difficult among the three exercises, and this can be a reason for the results observed also in the control group. Also executive functions seem to be improved by both adaptive and non-adaptive training, since both groups obtained better performance in the WCST after the treatment.

Moreover, results from FU assessment suggest a long-term effect only of the adaptive training on PwMS cognitive functions. In fact, 6 months after the end of the CR intervention, the ADAPT-gr maintained the improvement observed at POST in the PASAT-3 and SDMT, while the CONST-gr performance did not change across the time points.

It is worth noting that both groups understood the exercises and performed well along the training period, as shown by the high performance obtained by the CONST-gr and by the challenging difficulty levels maintained by the ADAPT-gr. Moreover, the amount of training was the same for the two groups, as reported by the adherence values. A possible explanation of our results is given by the theoretical framework according to which cognitive plasticity is driven by a prolonged mismatch between functional supplies and environmental demands [41]. A challenging task, resulting from an adaptive algorithm which set the difficulty level to the subject working threshold, can maximise the supply demand mismatch, thus enhancing the effect of training. Moreover, it was suggested that rehabilitation of cognitive processes that play central roles in the cognitive architecture and in brain areas that are active across a wide range of tasks [42] will maximise the applicability of the intervention effect (i.e., generality). WM seems to be one of the most prominent abilities in this regard [43], and this can explain why an adaptive training based on WM showed an effect in PwMS’ several cognitive domains functions.

Some limitations should be addressed to the present study. First, the two groups differed for one neuropsychological test at the baseline (i.e., SRT-LTS). In fact, the ADAPT-gr performed significantly worse than the CONST-gr before the training. However, the scores obtained at POST session by the ADAPT-gr were higher than those obtained by the CONST-gr. This could suggest some hidden effects of the adaptive paradigm also in verbal memory acquisition, which may be confused by the difference at baseline. Moreover, the FU assessment constituted of only two tests (i.e., PASAT-3 and SDMT). Although they are useful screening tools to measure CI in MS, a complete neuropsychological evaluation would have allowed for more consistent results on the treatment long-term efficacy. Further, we had some dropouts at FU. Even if no analysis option is ideal here, we did not exclude lost patients from analysis and we adopted the “last observation carried forward” method for the imputation of missing data, in order to respect the intention-to-treat principles. However, it is worth noting that the frequency of dropout did not differ between the treatment groups, thus not influencing the two-group comparison and suggesting that the number of dropouts did not depend on the kind of intervention received.

## Conclusion

In conclusion, an intensive WM training seems to be well accepted by PwMS and effective in improving their cognitive functions. Moreover, the results obtained in the present study suggest that an adaptive working load is a crucial feature determining the effectiveness of the intervention, allows a transfer effect also to non-trained cognitive domains and ensures a long-term positive effect. In our opinion, COGNI-TRAcK represents an optimal tool in order to administer a personalized training to cognitively impaired subjects, since the treatment characteristics can be tuned to the individual’s needs.

## Abbreviations

ADAPT-gr: Adaptive training group
ANOVA: Analysis of variance
BRB-NT: Rao’s Brief Repeatable Battery of Neuropsychological Tests
CI: Cognitive impairment
CONST-gr: Constant training group
CR: Cognitive rehabilitation
DMD: Disease-modifying drugs
Dual-Nback_task: “dual” *N*-back task
EDSS: Expanded disability status scale
MS: Multiple sclerosis
Oper-Nback_task: “Operation” *N*-back task
PASAT: Paced auditory serial addition test
PwMS: People with multiple sclerosis
RR: Relapsing-remitting
SD: Standard deviation
SDMT: Symbol digit modalities test
SP: Secondary progressive
SPART: 10/36 Spatial Recall Test
SPART-D: Spatial recall test-Delayed
SRT-CLTR: Selective reminding test-Consistent long term retrieval
SRT-LTS: Selective reminding test-Long term storage
SRT-D: Selective reminding test-Delayed
VS-WM_task: Visuospatial working memory task
WCST: Wisconsin card sorting test
WLG: Word list generation
WM: Working memory
